# DeepVIC: modular prediction and classification of bacterial virulence factors using protein language model embeddings

**DOI:** 10.1093/bioadv/vbag237

**Published:** 2026-08-17

**Authors:** Wai-Kai Tsui, You-Xiang Chan, Kin-Hung Chow, Pak-Leung Ho, Huiluo Cao

**Affiliations:** Department of Microbiology, University of Hong Kong, Hong Kong, 999077, China; Department of Microbiology, University of Hong Kong, Hong Kong, 999077, China; Department of Microbiology, University of Hong Kong, Hong Kong, 999077, China; Department of Microbiology, University of Hong Kong, Hong Kong, 999077, China; Carol Yu Centre for Infection, University of Hong Kong, Hong Kong, 999077, China; Department of Microbiology, University of Hong Kong, Hong Kong, 999077, China

## Abstract

**Motivation:**

Virulence factors (VFs) play critical roles in bacterial pathogenesis, and identifying these proteins and elucidating their mechanisms is essential for developing effective infection treatments. While protein language models (PLMs) have revolutionized the protein analysis, current *in silico* methods for classifying VFs primarily rely on fine-tuned PLMs ProtBert-BFD that perform identification and classification in a single step. This monolithic approach lacks flexibility and is vulnerable to error propagation, particularly from false negatives. Moreover, the direct use of PLM embeddings as features for VF prediction and classification remains underexplored.

**Results:**

To overcome these limitations, we introduce the Deep learning VF Identifier and Classifier (DeepVIC), a modular framework that separates identification and classification into distinct states. The identification module employs a binary classifier using information-rich PLM embeddings. The classification module then integrates these embeddings with evolutionary features derived from position-specific scoring matrices (PSSMs) in a multiclass classifier, structured according to the virulence factor database (VFDB) schema. We benchmarked DeepVIC against six state-of-the-art VF classifiers and predictors using a large independent holdout dataset and two literature-curated datasets for *Streptococcus pneumoniae* and *Lactococcus* spp. DeepVIC demonstrated strong generalizability, achieving robust and balanced performance on the independent holdout dataset and competitive recall of putative VFs from existing literature. The modular, task-separation architecture adopted in DeepVIC offers exceptional flexibility, making it a uniquely adaptable and competitive framework. Model interpretability strategies reveal that DeepVIC leverages biologically relevant motifs and effectively integrates PLM embeddings with evolutionary features, offering insights into how deep learning models interpret VFs.

## 1 Introduction

Bacterial pathogens employ diverse mechanisms to cause disease in humans, with virulence factors (VFs) playing a crucial role in host invasion, colonization and pathogenicity (Rasko and Sperandio 2010, Liu *et al*. 2022, Qin *et al*. 2022, Sharma *et al*. 2022). While some VFs have been well-characterized (Granum and Lund 1997), those in emerging or understudied pathogens, such as *Lactococcus* spp., remains poorly understood (Chan *et al*. 2024). VFs encompass a broad spectrum of structural and molecular features, from toxins to structural proteins (Chen *et al*. 2023, Nikolic and Mudgil 2023), complicating their identification. Elucidating these factors is essential for understanding pathogenic mechanisms and developing targeted treatments.

Traditional VF identification relies on resource-intensive experimental approaches such as mutagenesis, *in vitro* and *in vivo* assays. In contrast, computational methods offer rapid and cost-effective alternatives for preliminary screening of candidate VFs. Alignment-based methods identify VFs through sequence similarity using tools like the basic local alignment search tool (BLAST) (Altschul *et al*. 1990), ultra-fast search and clustering (USEARCH) (Edgar 2010), the BLAST-like alignment tool (BLAT) (Kent 2002), and double index alignment of next-generation sequencing data (DIAMOND) (Buchfink *et al*. 2015) for pairwise alignment, or employ hidden Markov models (HMMs) for multi-sequence alignment (MSA) (Eddy 1998). While effective for detecting known VFs, these methods show limited performance for novel pathogens and suffer from high false-positive rates in HMM-based searches, along substantial computational costs for profile construction. Furthermore, their effectiveness is constrained by the coverage limitations of existing VF databases including MvirDB (Zhou *et al*. 2007), VFDB 2025 (Zhou *et al*. 2025), PATRIC (the Bacterial and Viral Bioinformatics Resource Center, BV-BRC) (Olson *et al*. 2023), VICTORS (Sayers *et al*. 2019), and VFNet datasets computationally expanded from VFDB (Zheng *et al*. 2020).

The integration of machine learning has significantly advanced VF prediction. Early tools primarily utilized support vector machine (SVM), including VirulentPred (Garg and Gupta 2008) and its successor (Sharma *et al*. 2023), as well as VICMpred (Saha and Raghava 2006). MP4 utilizes SVM in its algorithm while its predecessor MP3 combined HMM with SVM (Gupta *et al*. 2014, 2022). More sophisticated methods like PathoFact integrated HMM with random forests and incorporated a secretion prediction module using SignalP (de Nies *et al*. 2021). Recent advances have employed deep learning models that leverage abundant sequence-derived features (Xie *et al*. 2020, Singh *et al*. 2024) and k-mer representations [e.g. PreVFs-RG (Zhang and Jing 2023)], though one-hot encoding remains largely limited to simple deep neural networks (DNN) (Ji *et al*. 2023).

A transformative development emerged with protein language models (PLMs), which excel at capturing amino acid physiochemical properties (Rives *et al*. 2021) and generate information-rich embeddings suitable for diverse applications including sequence alignment (Liu *et al*. 2024, Pantolini *et al*. 2024), functional annotation, protein classification (Pokharel *et al*. 2022, Flamholz *et al*. 2024), and physical property prediction (Xiong *et al*. 2024, Zhang *et al*. 2024). These capabilities have been successfully adapted for VF prediction through several approaches: FunGeneTyper (Zhang *et al*. 2024) and PLMVF (Li *et al*. 2025) implements the Evolutionary Scale Modelling ESM-1b and ESM-2, respectively (Rives *et al*. 2021, Lin *et al*. 2023), DTVF (Sun *et al*. 2024) utilizes ProtT5 PLM embeddings, and GTAE-VF (Li *et al*. 2024) employs a Graph Neural Network trained on ESMFold (Lin *et al*. 2023). Despite these advancements, current PLM-based tools remain primarily focused on binary VF prediction, with classification receiving less attention. Even tools trained from same training data, like VFNet (Zheng *et al*. 2020) and FunGeneTyper (Zhang *et al*. 2024), exhibit substantial variation in their classification schemas. While VirulentHunter has adapted ESM-2 for fine tuning and classifies VFs according to the 14 VF classes in VFDB (Chen *et al*. 2025), utilizing PLM embeddings as prominent features in VF classification remained unelucidated. Previous works on VF prediction and VF databases are summarized in Table S1A and S1B, respectively. Additionally, the inherent opacity of these models continues to limit interpretability, though emerging techniques such as Shapley values (Lundberg and Lee 2017) and integrated gradients (Sundararajan *et al*. 2017) are helping to address this fundamental limitation.

**Figure 1:**
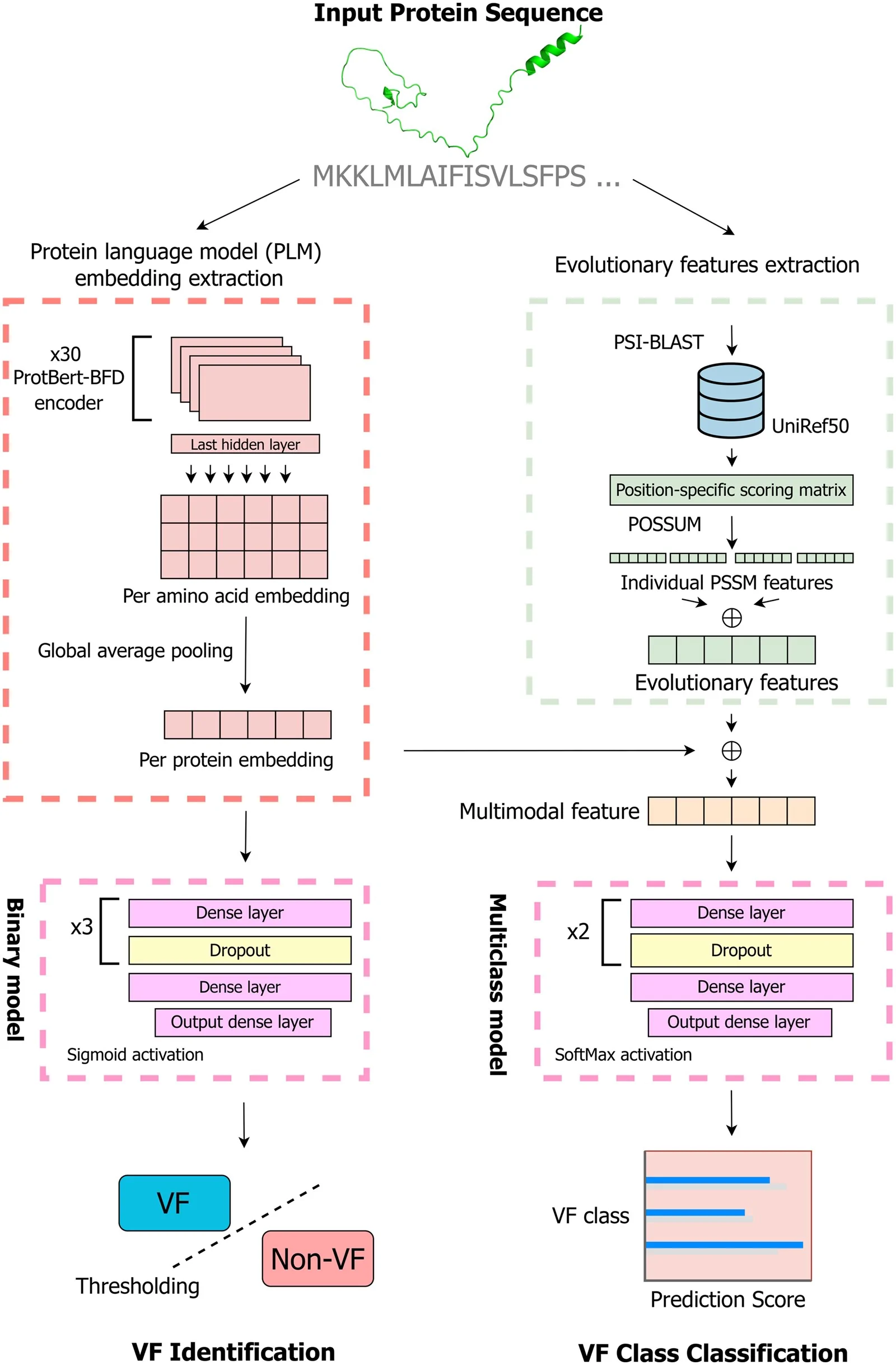
Schematic overview of the DeepVIC workflow from data processing to model prediction.

To address these challenges, we developed the Deep learning Virulence factor Identifier and Classifier (DeepVIC), which integrates PLMs ProtBert Big Fantastic Database (BFD) embeddings (Elnaggar *et al*. 2022) with evolutionary features derived from Position-Specific Scoring Matrices (PSSM) while adopting the standardized VFDB classification schema. DeepVIC incorporates model interpretability strategies to enhance biological interpretability of model decision-making. The complete workflow and architecture of the model are illustrated in Fig. 1.

## 2 Methods

### 2.1 Dataset curation and pre-processing

VFs were mainly retrieved from PATRIC (Olson *et al*. 2023), VICTORS (Sayers *et al*. 2019), VFDB (Liu *et al*. 2022), UniProtKB/Swiss-Prot (Consortium 2022) on 27 November 2023. For UniProtKB, sequences annotated with the ‘virulence’ keyword (KW-0843) from bacteria (Taxonomy ID: 2) comprised the positive dataset, while those without served as negatives (Fig. S1A and B). The collective datasets underwent rigorous quality control, beginning with sequencing clustering using CD-HIT v4.8.1 (Fu *et al*. 2012) at a 0.7 identity threshold, as used in other studies (Boopathi *et al*. 2019, Collatz *et al*. 2021, Shi *et al*. 2024). To assess the effect of sequence redundancy, additional clustering-threshold sensitivity analyses were performed at 0.3, 0.4, 0.5, 0.6 and 0.7 identity thresholds. Deduplication with SeqKit v2.6.1 (Shen *et al*. 2016) and BLASTP v2.9.0 at 40% identity and 80% query coverage was followed to eliminate potential VFs in the negative dataset.

For the binary classifier, to balance with the positive dataset, negative sequences were randomly selected using seqtk v1.4 (https://github.com/lh3/seqtk) (2023). The dataset was split into training and independent test dataset at an 8:2 ratio, stratified by binary labels. For the multiclass classifier, the positive dataset with assigned class types (*n* = 12 989) was partitioned into training (60%), validation (20%), and independent holdout test (20%) sets, maintaining stratification by VFDB class assignments. Proteins not originating from VFDB were assigned classes through BLASTP at 40% identity and 70% query coverage threshold. To address class imbalance in the multiclass training set, we applied the SMOTE algorithm from imbalanced-learn v0.11.0 (Chawla *et al*. 2002).

### 2.2 Feature extraction

Per-protein embeddings were created by global average pooling with ‘max_length’ parameter set at 2000 using PLM ProtBert Big Fantastic Database (BFD) (Elnaggar *et al*. 2022), with the max_length parameter set to 2000. ProtBert-BFD was selected as the default PLM representation because it has been widely used as a frozen feature extractor in protein functional annotation tasks (Pokharel *et al*. 2022, Flamholz *et al*. 2024), and provides longer sequence coverage under our implementation. This is relevant for VF prediction because many VFs are large multidomain proteins PSSM evolutionary features listed in Table S2 were extracted using POSSUM (Wang *et al*. 2017) after searching against the UniRef50 database using PSI-BLAST v2.9.0 (Suzek *et al*. 2015). Sequences with no PSI-BLAST hits were assigned zeroes for feature vectors. Feature selection process excluded length-dependent features, composite features (AADP-PSSM, AATP, MEDP), and Tri-Gram PSSM due to its disproportionate vector size (8000 dimensions) that could dominate other features.

To evaluate the potential benefits of structural enriched sequence representations, we conducted comparative experiments using embeddings from the Implicit Structure Model (ISM) PLM (Ouyang-Zhang *et al*. 2024), which has demonstrated effectiveness in bacterial peptide classification (Cordoves-Delgado and García-Jacas 2024). These embeddings were similarly processed through global average pooling, with sequences exceeding 1022 amino acids truncated to accommodate the input-length limitation of ISM.

To visualize the distribution of the entire VF dataset (*n* = 33 456), fused feature vectors (ProtBert-BFD PLM + 6 optimal evolutionary features) were projected into two dimensional space using UMAP v0.5.6 (McInnes *et al*. 2018) with n_neighbors = 50 and min_dist = 0.5. Cosine similarities of these feature vectors between VFs were calculated to assess intraclass and interclass relationships.

### 2.3 Model architecture

DeepVIC employs a DNN architecture with dropout regularization (rate = 0.2) for both classification tasks. The binary classifier consists of four fully connected layers (512, 256, 64, and 8 neurons, respectively) with a sigmoid output unit, while the multiclass classifier uses three layers (512, 256, and 128 neurons) with a softmax output layer (Flamholz *et al*. 2024), corresponding to the 14 major VFDB classes. Both models were trained with batch size 64 and learning rate 0.001, with the multiclass model incorporating early stopping (patience = 5 epochs).

### 2.4 Model optimization and evaluation

A comprehensive evaluation strategy was implemented beginning with baseline comparisons between models trained on one-hot encoded sequences (Supplementary Method S1) and ProtBert-BFD PLM embeddings for the binary classifier. Both models used hyperparameters (batch size = 64, learning rate = 0.001 and epoch = 15), with the one-hot encoded model incorporating an additional flatten layer after the input layer. Model performance was evaluated using Area Under the Receiver Operating Characteristic Curve (AUROC) with 5-fold cross-validation.

Following the demonstration of superior performance with PLM embeddings in baseline comparisons, we performed a rigorous 5*5-fold nested cross-validation to evaluate the binary classification model. The inner folds were used to select hyperparameters based on average AUROC, while the outer folds provided an unbiased performance evaluation of the final model. Final tested model hyperparameters were retrieved from a further 5-fold cross-validation referring to the highest average AUROC. Along with these hyperparameters (Table S3), the maximum Youden’s *J* statistic in each fold was retrieved by evaluating over all true positive rates (TPR) and false positive rates (FPR) across data points. Youden’s *J* statistic (Youden 1950) is defined as:


J=sensitivity+specificity−1

where


$$\begin{array}{c} \mathrm{sensitivity}=\mathrm{TPR}\\ \text{specificity}=1-\mathrm{FPR} \end{array}$$

TPR and FPR are defined as:


$$\begin{array}{c} T\mathrm{PR}=\frac{\mathrm{TP}}{\mathrm{TP}+\mathrm{FN}}\\ \text{FPR}=\frac{\text{FP}}{\text{FP}+\text{TN}} \end{array}$$

where TP, TN, FP, FN corresponds to true positives, true negatives, false positives, false negatives in the predicted labels respectively. The resulting formula can be presented as:


$$\begin{array}{c} J=\text{TPR}+(1-\text{FPR})-1\\ J=\text{TPR}-\text{FPR} \end{array}$$

The optimal cutoff was determined by averaging the maximum *J* statistic values across folds, which corresponds to the point on the Receiver Operating Characteristic (ROC) curve furthest from random guessing line. The binary model was finally trained using the entire training data and selected hyperparameters.

For development of the multiclass classifier, we began evaluation with a 3-layer DNN architecture using a batch size = 64, learning rate = 0.001, and incorporated early stopping patience = 5. Given the computational complexity of simultaneous optimization, we adopted a two-stage strategy as illustrated in Fig. S2. First, we performed feature selection by concatenating of PSSM-derived evolutionary feature vectors with PLM embeddings and conducting grid search to identify optimal feature combinations using baseline hyperparameters. Second, we fine-tuned the batch size, learning rate, and early stopping patience through additional grid search. At each optimization step, we selected models achieving the highest weighted F1 score on the validation data. The tested hyperparameter combinations are presented in Table S3.

The binary and multiclass models were finally evaluated on the respective independent holdout datasets (*n *= 2598). The binary classifier was evaluated using accuracy, F_1_ score, AUROC, Area Under Precision Recall Curve (AUPRC), and Matthews correlation coefficient (MCC), while the multiclass VF classifier was evaluated using accuracy, weighted F_1_ score, and micro-average AURPC. They are defined as:


$$\begin{array}{c} \mathrm{Accuracy}=\frac{\mathrm{TP}+\mathrm{TN}}{\mathrm{TP}+\mathrm{TN}+\mathrm{FP}+\mathrm{FN}}\\ \begin{array}{c} \text{Precision}=\frac{\text{TP}}{\text{TP}+\text{FP}}\\ \begin{array}{c} \text{Recall}=\frac{\text{TP}}{\text{TP}+\text{FN}}\\ \begin{array}{c} F_{1}=\frac{2\times \text{Precision}\times \text{Recall}}{\left(\text{Precision}+\text{Recall}\right)}\\ \text{MCC}=\frac{\text{TP}\times \text{TN}-\text{FP}\times \text{FN}}{\sqrt{\left(\text{TP}+\text{FP})(\text{TP}+\text{FN})(\text{TN}+\text{FP})(\text{TN}+\text{FN}\right)}} \end{array} \end{array} \end{array} \end{array}$$

All model evaluation metrics were implemented using the scikit-learn v1.4.2 package.

### 2.5 Additional robustness and benchmarking analyses

To further evaluate the robustness and fairness of DeepVIC, we performed four additional analyses, with full details provided in Supplementary Method S2. First, to assess the influence of sequence redundancy, DeepVIC was retrained using datasets clustered at CD-HIT identity thresholds of 30%, 40%, 50%, 60%, and 70% and evaluated using AUROC, F1-score, accuracy, and MCC. Second, to examine the effect of realistic class imbalance, we trained DeepVIC under VF: non-VF ratios of 1:1, 1:2, 1:5, 1:10, and 1:20 and assessed validation-based threshold calibration. Third, DeepVIC was benchmarked against representative traditional machine learning and neural network models, including XGBoost, Random Forest, SVM, and 1D-CNN, using the same dataset split and feature representation. Finally, to ensure fair comparison with existing VF predictors, available SOTA tools were retrained or reimplemented using the same DeepVIC training dataset and evaluated on the same independent test set.

### 2.6 Generalizability evaluation of DeepVIC

To demonstrate generalizability, DeepVIC was evaluated on curated VF sets from *Streptococcus pneumoniae* (Table S4) (Cao *et al*. 2021) and *Lactococcus* spp. (Table S5) (Chan *et al*. 2024). To prevent data leakage and metrics inflation, putative VF sequences with 100% identity, 100% coverage with the training dataset were excluded (38/150 for *S. pneumoniae*, 5/68 for *Lactococcus* spp.). Recall rates of the putative VFs were compared against 6 state-of-the-art (SOTA) VF predictors, which are either online (MP4 and DeepVF) or readily accessible programs (DTVF, FunGeneTyper, VirulentHunter, and VirulentPred 2.0), and BLASTP on the training data (80% identity, 70% coverage, *E*-value < 1e − 10) for the two curated VF datasets. Existing VF prediction tools were used as pre-trained models with their default parameters and original weights, without retraining on our dataset, reflecting real-world usage by end-users. This analysis was considered a sensitivity-oriented external usability comparison because the curated datasets contain only VF sequences. Separately, a fair retraining benchmark using the same DeepVIC training and independent test sets was performed as described above. BLASTP was run against the DeepVIC training dataset as the reference database. Except for DeepVF adopting a cutoff of 0.85 (Xie *et al*. 2020), a cutoff of 0.5 was used in other predictors.

Additionally, 73 *S. pneumoniae* serogroup 24 genomes were annotated using Prokka v1.14.6 (Seemann 2014, Cao *et al*. 2021) (Table S6). Protein sequences with BLASTP hits (80% identity, 70% coverage, *E*-value < 1e − 10) against the 150 putative *S. pneumoniae* VFs were evaluated using DeepVIC.

### 2.7 Model interpretation

Feature importance were assessed using SHAP values computed via shap. DeepExplainer (Lundberg and Lee 2017), with 1000 randomly selected training sequences as background.

Domain regions were identified using InterProScan v5.69–101.0 (Jones *et al*. 2014), and perturbation tests were performed by masking these regions with “X” residues. Protein structures and motifs were visualized using coordinates obtained from domain annotations (Supplementary Method S3).

### 2.8 Statistical analysis and plotting

All statistical analysis were implemented using SciPy v1.13. Data normality was determined by Shapiro-Wilk tests. For performance comparisons, Student’s *t*-tests were applied with Nadeau-Bengio correction (Nadeau and Bengio 2003, Bouckaert and Frank 2004) to account for cross-validation dependencies. For multiclass classifier interpretability evaluation, Kruskal-Wallis *H* tests with Dunn’s post-hoc analysis (Holm-Bonferroni corrected) were performed to compare differences of SHAP values across VF classes. Visualizations were generated with matplotlib v3.9.2 and seaborn v0.13.2.

### 2.9 Hardware specifications

All training and validation were performed on a workstation equipped with an NVIDIA RTX A6000 GPU, AMD Ryzen Threadripper PRO 5945WX CPU, and 128 GB of RAM.

## 3 Results

### 3.1 Training dataset characterization

A comprehensive dataset of 33 456 verified VF protein sequences was compiled from PATRIC, VICTORS, VFDB, and UniProtKB, representing the largest training dataset among current VF predictors. Of these, 12 989 (38.8%) were annotated with VFDB class labels across 14 functional categories: “adherence,” “invasion,” “effector delivery system,” “motility,” “exotoxin,” “exoenzyme,” “immune modulation,” “biofilm,” “nutritional/metabolic factor,” “stress survival,” “regulation,” “post-translational modification,” “antimicrobial activity/competitive advantage,” and “others.” The dataset exhibited notable class imbalance, with “effector delivery system” representing 32.8% of sequences assigned, compared to only 0.18% for “antimicrobial activity/competitive advantage” (Fig. S3A). Sequence length analysis revealed that 70.2% and 86.4% of VFs were ≤1000 and ≤2000 amino acids, respectively (Fig. S3B). Taxonomic distribution showed *Pseudomonadaceae* as the most represented family (7.2%), with pathogenic genera including *Mycobacterium*, *Yersinia*, *Bacillus*, and *Escherichia* also well represented (Fig. S3C and D). For the binary classifier, an equal number of negative (non-VF) sequences were randomly selected, resulting in a balanced dataset of 66 912 total sequences, which was split 80:20 into training (*n* = 53 528) and independent test (*n* = 13 384) sets. For the multiclass classifier, the 12 989 VFs with VFDB class annotations were partitioned into training (60%, *n* = 7793), validation (20%, *n* = 2598), and independent test (20%, *n* = 2598) sets, maintaining stratification by VFDB class.

**Figure 2:**
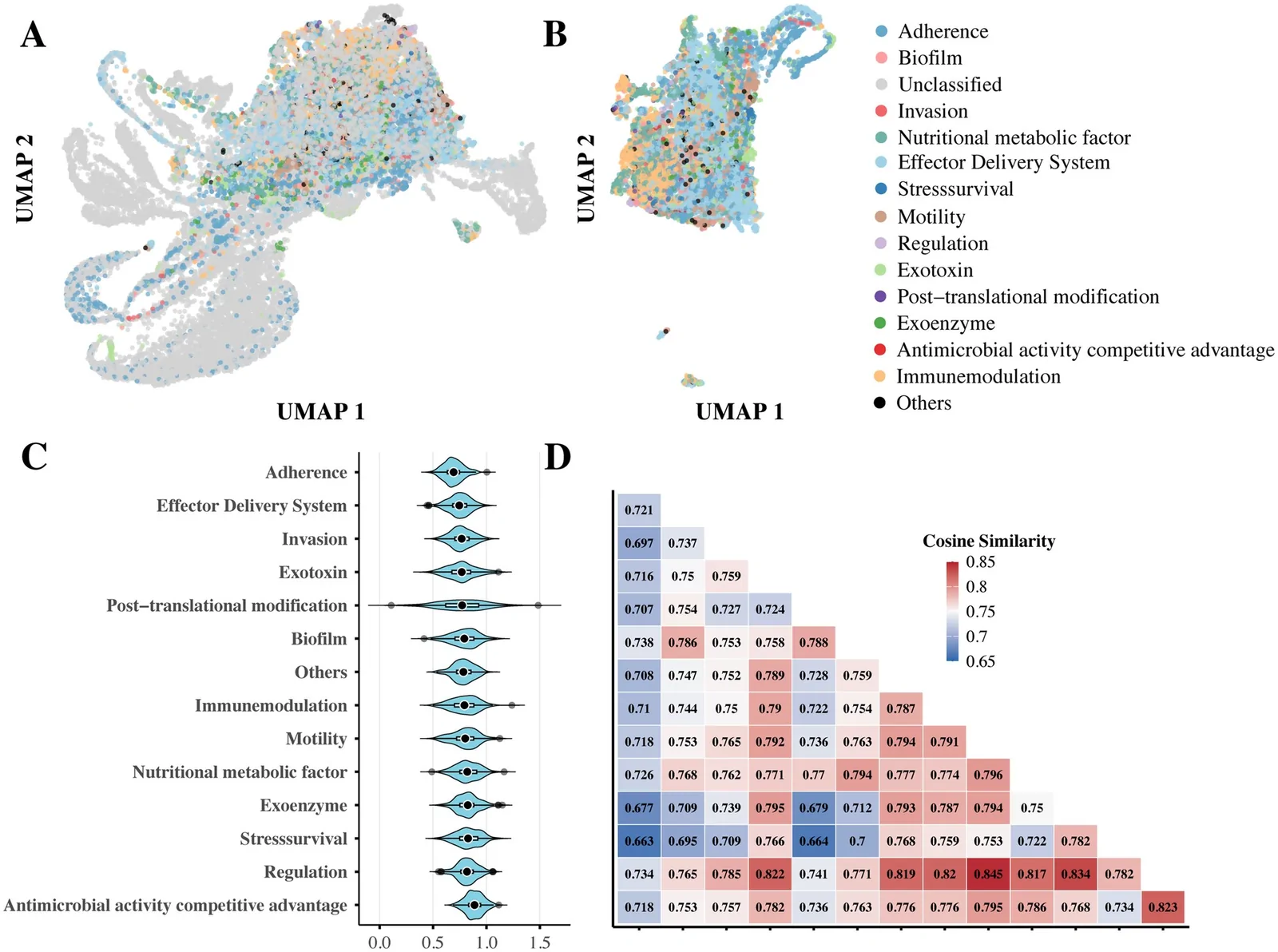
Distribution and similarity structure of VF feature representations. UMAP projection of the fused feature vector (PLM embedding with 6 optimal PSSM evolutionary features) for (A) the entire positive dataset and (B) the dataset only with assigned VFDB classes with assigned VFDB VF class in color. (C) Violin plot showing pairwise intraclass cosine similarities among VF classes. (D) Heatmap of mean pairwise interclass cosine similarities between different VF classes.

Uniform manifold approximation and projection (UMAP) visualization revealed distinct clustering patterns between classified and unclassified VFs (Fig. 2A). Sequences annotated with VFDB classes formed distinct clusters, whereas unclassified VFs grouped separately. However, separation between different VF classes was less pronounced. Certain classes, such as “immune modulation,” “exotoxin,” and “stress survival,” exhibit well-defined clusters while others, such as “adherence” and “effector delivery system,” showed dispersed and less cohesive clustering patterns (Fig. 2B). The distinct clustering patterns of feature vectors highlight that VF classification could be feasible with our selected features.

For sequences with assigned class labels, cosine similarity was examined. The “antimicrobial activity/competitive advantage” class exhibited a narrower range of intraclass cosine similarity, and a higher mean interclass cosine similarity compared to other VF classes (Fig. 2C and D and Table S7). No statistically significant relationships were observed between sequence abundance and mean cosine similarity (Fig. S3E).

### 3.2 Binary classifier performance

From 5-fold cross-validation, the baseline model achieved a mean AUROC of 0.858 (95% CI: 0.854–0.862) with one-hot encoding and 0.943 (95% CI: 0.940–0.947) with PLM embedding. PLM embeddings were selected for downstream cross-validation and model building due to their statistically superior performance (*P *< .001) (Table S8).

**Figure 3:**
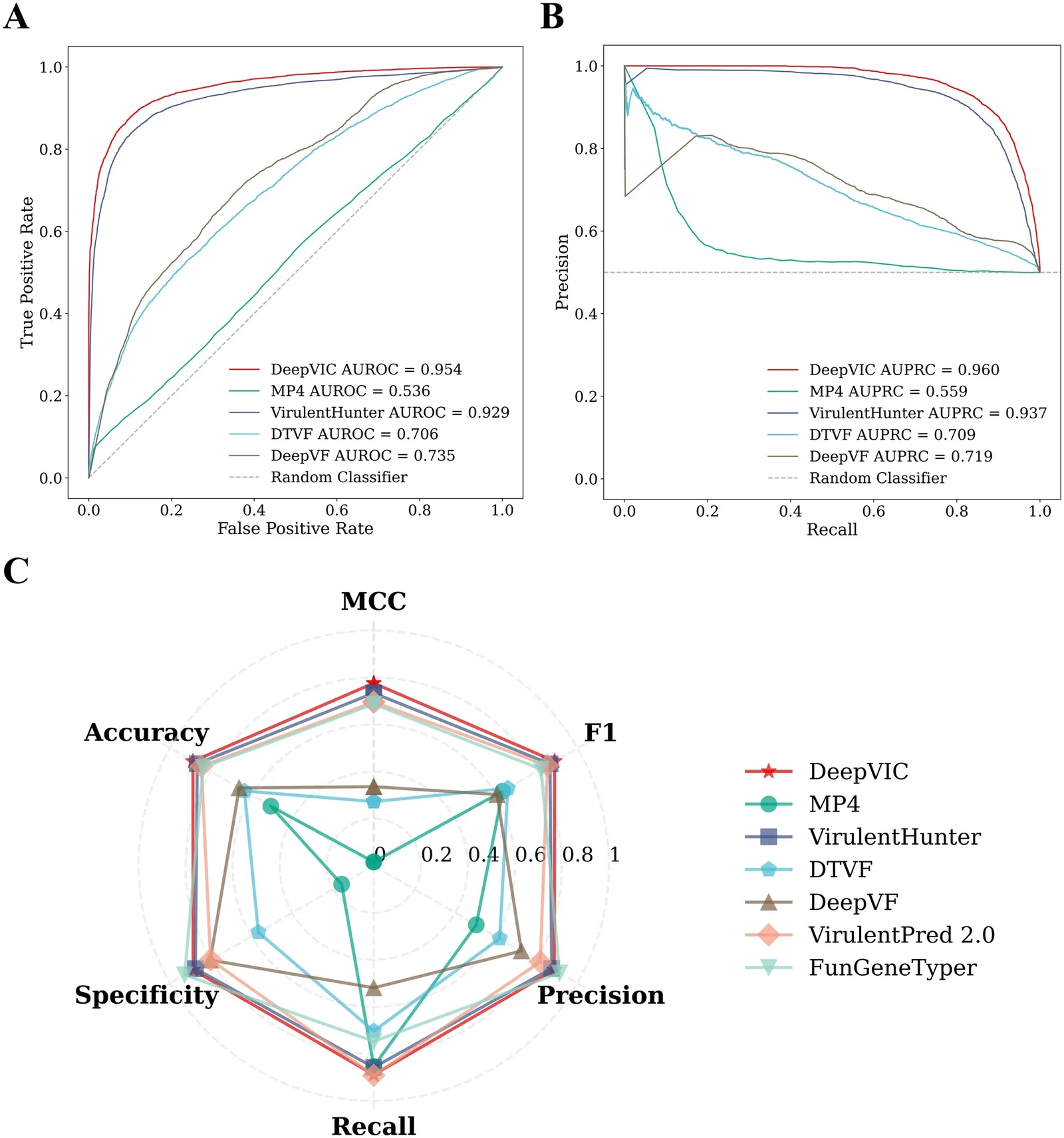
Performance comparison of the DeepVIC binary model against other available VF predictors on an independent holdout dataset. (A) Receiver operating charactersitic and (B) precision-recall curves (excluding VirulentPred 2.0 and FunGeneTyper, which lack prediction scores). (C) Radar chart comparing key performance metrics across DeepVIC binary model and all six VF predictors.

In nested cross-validation, the binary classifier model achieved a mean AUROC of 0.950 (95% CI: 0.946–0.953). The optimal cutoff, determined by maximizing Youden’s *J* statistic in the 5-fold cross-validation, was 0.537 and was applied to the final binary classifier. DeepVIC demonstrated robust performance on the independent holdout dataset (*n *= 13 383 positive labels = 6691, negative labels = 6692) (Table S9), with an F_1_ score of 0.888, an AUROC of 0.954 slightly outperforming the nested cross-validation mean AUROC (*P *= .011) (Table 1), an AUPRC of 0.960, a MCC of 0.776, and an accuracy of 88.8%. The ROC curve and precision-recall (PR) curves of DeepVIC’s binary model showed significant deviation from random classifiers (Fig. 3A and B).

**Table 1:** Performance metrics of DeepVIC binary classifier across evaluation datasets, showing results from 5-fold nested CV^a^ and the independent holdout test.

	Nested 5-fold CV^a^	Independent dataset
AUROCs^b^	0.953	0.954
	0.951	
	0.946	
	0.948	
	0.950	
Mean AUROC^b^	0.950	
95% CI^c^	0.946 – 0.953	
Shapiro-Wilk *P*	.997	
Corrected one sample t-test *P*	.011	

a CV, cross-validation.

b AUROC, area under receiver operating characteristic curve.

c CI, confidence interval.

To evaluate DeepVIC’s binary model, we benchmarked it against six SOTA predictors on an independent dataset. DeepVIC outperformed the six models in overall accuracy, F_1_, and MCC (Fig. 3C and Table S10), indicating the best-balanced performance. This result was further corroborated by the higher areas under ROC and PR curves (Fig. 3A and B), although VirulentPred 2.0 and FunGeneTyper were excluded in this comparision as they do not report prediction scores. While FunGeneTyper showed the highest precision and specificity, and VirulentPred 2.0 attained the highest recall, DeepVIC’s strong performance across all metrics confirms its robustness as a general-purpose predictor.

To further evaluate DeepVIC’s binary model, we performed four additional analyses. First, to address potential concerns about performance inflation due to sequence redundancy, we re-trained DeepVIC across CD-HIT clustering thresholds ranging from 30% to 70%. Stricter clustering substantially reduced the number of retained sequences. At the 30% threshold, 16 237 sequences were retained, corresponding to 24.3% of the original binary dataset, whereas the 70% threshold retained 64 461 sequences, corresponding to 96.3% retention. Performance decreased gradually under more stringent clustering but remained robust: at 30% identity, DeepVIC achieved AUROC = 0.918, F1-score = 0.820, accuracy = 0.837, and MCC = 0.676, compared with AUROC = 0.949, F1-score = 0.871, accuracy = 0.881, and MCC = 0.765 at 70% identity (Table S11). These results indicate that sequence redundancy may contribute modestly to performance, but DeepVIC is not solely dependent on highly similar sequences.

Second, to evaluate robustness under more realistic class distributions, we performed imbalance-sensitivity analyses using VF: non-VF ratios from 1:1 to 1:20. These experiments showed that balanced training remains appropriate for the final DeepVIC binary model because it avoids majority-class dominance and maintains balanced sensitivity and specificity. Validation-based threshold calibration further improved F1-score and MCC under imbalanced settings, supporting threshold adjustment when applying DeepVIC to datasets with different VF prevalence (Table S12).

Third, we benchmarked DeepVIC against representative traditional machine learning and neural network models, including XGBoost, Random Forest, SVM, and 1D-CNN, using the same dataset split and input feature representation. DeepVIC achieved the highest mean AUROC and MCC among these models, while 1D-CNN achieved comparable accuracy and slightly higher F1-score. XGBoost also performed competitively but showed slightly lower F1-score and MCC than DeepVIC (Table S13).

Finally, to ensure fair comparison with existing VF predictors, we retrained or reimplemented available SOTA tools using the same training dataset used for DeepVIC and evaluated all models on the same independent test set (Table S14). DeepVIC achieved the highest F1-score, MCC, AUROC, precision, and recall among the evaluated models, with F1-score = 0.94813, MCC = 0.87599, AUROC = 0.98236, precision = 0.95700, and recall = 0.92925. MP4, DTVF, FunGeneTyper-style classifier, DeepVF-style stacked model, VirulentHunter, and VirulentPred-style model achieved AUROC values of 0.92133, 0.93044, 0.95616, 0.98119, 0.97406, and 0.93090, respectively [1–6]. Although the DeepVF-style stacked model achieved higher accuracy and specificity, DeepVIC showed the best overall discriminative performance based on F1-score, MCC, AUROC, precision, and recall.

### 3.3 Multiclass classifier performance

**Figure 4:**
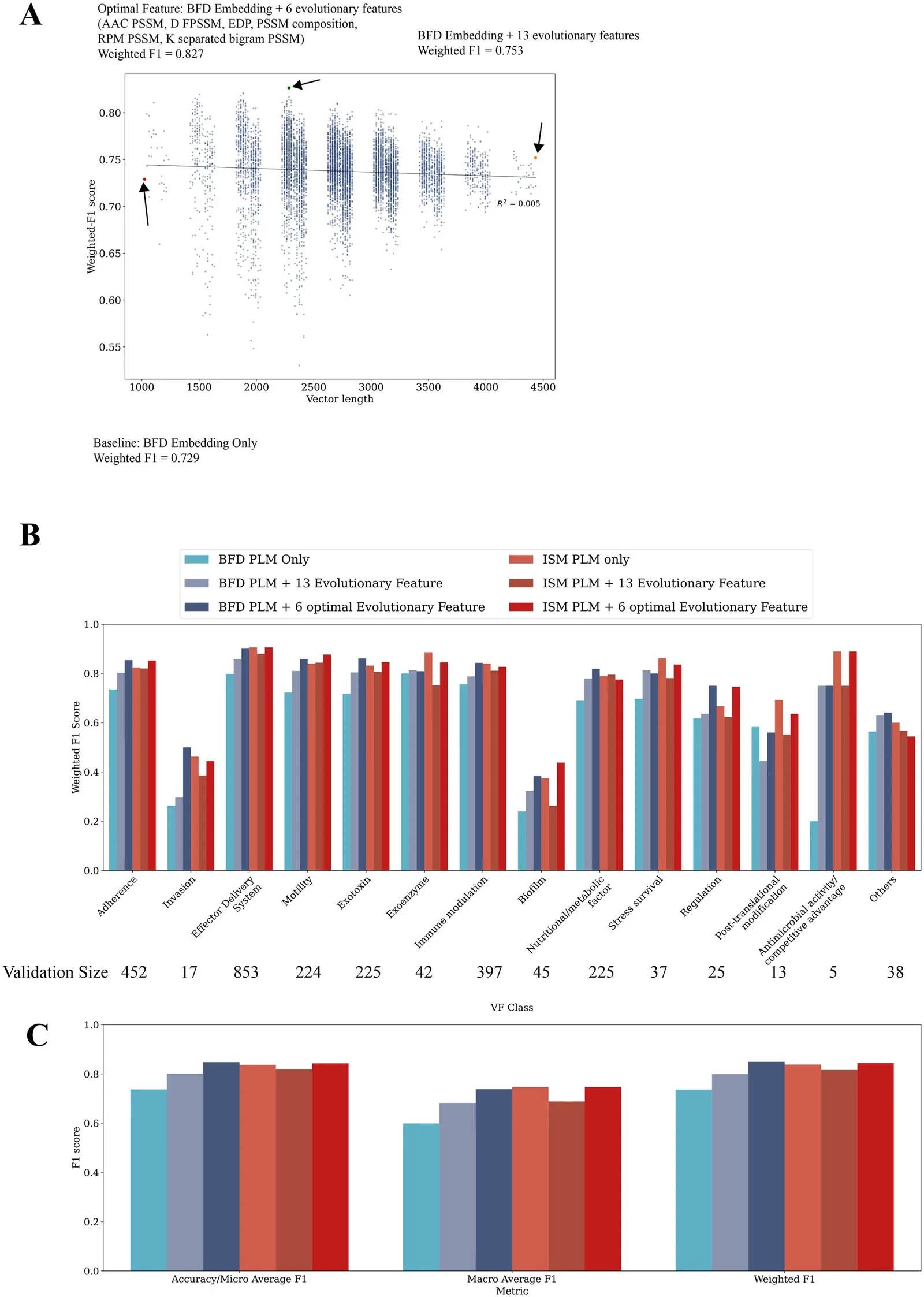
Feature optimizations for the multiclass classifier model. (A) Scatter plot comparing vector size from grid search combinations against weighted F_1_ scores using cross-validation dataset with baseline hyperparameters. Red and orange points represent models’ performance using either ProtBert-BFD embeddings alone or combined with all 13 evolutionary features, while the green point represents the optimized performance. (B) VF class-specific performance and (C) overall model performance across different feature combinations.

The initial model using ProtBert-BFD PLM embeddings with baseline hyperparameters yielded suboptimal performance (overall accuracy: 72.9%; weighted F_1_ score: 0.729). To enhance predictive power, a grid search identified an optimal combination of evolutionary features derived from PSSMs, including amino acid composition, D-FPSSM, PSSM composition, EDP from evolutionary difference PSSM, probe microarray RPM-PSSM, and K-separated bigram PSSM. Scatterplot comparing feature vector combinations revealed that while adding evolutionary features to PLM embeddings alone provided no significant improvements (Fig. 4A), the combination of ProtBert-BFD PLM embeddings with six optimal evolutionary features (PLM6E) demonstrated superior performance to both the baseline model and the model incorporating all 13 evolutionary features in the validation dataset (Fig. 4B and C and Table S15).

**Figure 5:**
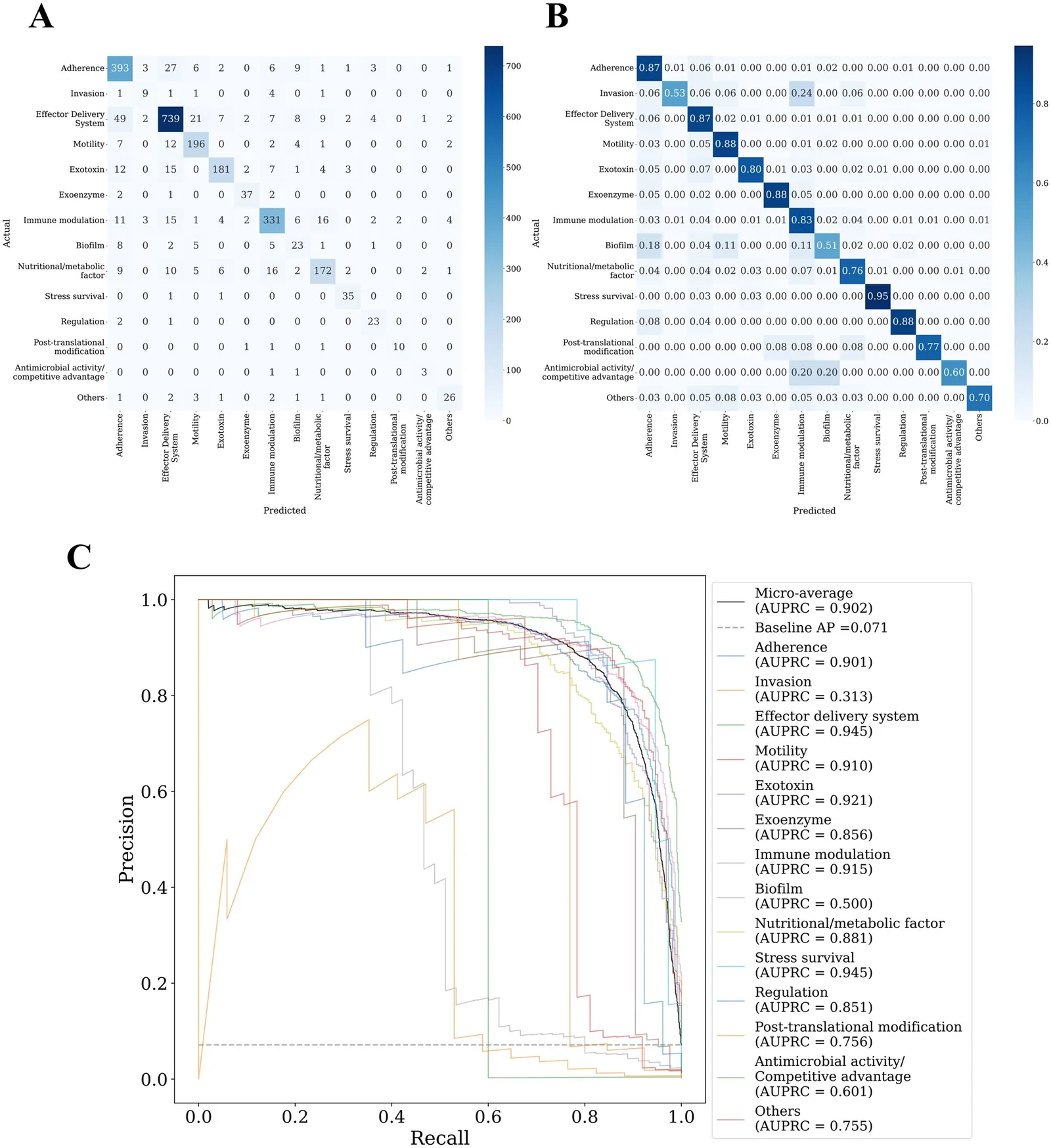
Multiclass classifier evaluation on the independent holdout dataset. (A) Confusion matrix showing raw prediction counts across VF classes. (B) Normalized confusion matrix showing prediction relative to each true VF class. (C) Precision-recall curves for 14 VF classes with micro-average precision-recall curve. Dotted diagonal line represents random guessing.

The PLM6E model was selected for subsequent analyses, and it demonstrated strong generalizability, with consistent performance improvements across most VF classes in both the validation dataset and independent holdout datasets (Table 2). However, VF classes with limited data (e.g. “invasion” and “biofilm”), showed persistently lower performance in validation dataset (Fig. 4B). This finding was corroborated by confusion matrices of the independent holdout dataset, which revealed strong predictive accuracy for most classes but poorer performance for these underrepresented categories (Fig. 5A–C). Substitution of ProtBert-BFD embeddings with structurally enriched ISM embeddings yielded comparable overall performance metrics (Fig. 4B and C and Table S15), The difference between ISM- and ProtBert-BFD-based models was not statistically significant. ProtBert-BFD combined with the six selected evolutionary features achieved the best aggregate performance in Fig. 4C and the best class-specific F1 score in 7 out of 14 VF classes, including several large classes. Therefore, ProtBert-BFD was retained as the default representation because it provided comparable performance, longer sequence coverage under our implementation, and consistency across the binary and multiclass modules.

**Table 2:** Comparative performance of the DeepVIC multiclass classifier on validation and independent test datasets.

Metric	Validation dataset	Independent dataset
Accuracy	0.848	0.838
Weighted Average F1	0.849	0.839
Macro Average F1	0.738	0.758
F1 score by VF class		
Adherence	0.854	0.830
Invasion	0.5	0.529
Effector Delivery System	0.903	0.880
Motility	0.858	0.848
Exotoxin	0.861	0.848
Exoenzyme	0.809	0.860
Immune modulation	0.843	0.848
Biofilm	0.383	0.46
Nutritional/metabolic factor	0.818	0.796
Stress survival	0.8	0.875
Regulation	0.75	0.780
Post-translational modification	0.56	0.8
Antimicrobial activity/competitive advantage	0.75	0.545
Others	0.641	0.712

To investigate factors influencing model performance, we examined the relationships between number of sequences in VF classes, the mean intra-class cosine similarity, and VF class specific weighted F_1_ scores. Contrary to previous reports (Zheng *et al*. 2020), we found no statistically significant correlation between cosine similarity and F_1_ scores (Fig. S1F). However, we observed a statistically significant nonlinear positive relationship between sequence abundance and F_1_ scores (Fig. S1G), following an inverse power law. This suggests that sequence abundance within a VF class is a stronger determinant of class-specific performance than cosine similarity, but performance improvements diminish at higher sequence abundance (Fig. S1G).

Performance of the DeepVIC multiclass model was also compared against SOTA tools on the independent dataset. A direct comparison was only feasible with VirulentHunter, as it shares the same VFDB classification schema. For a fair assessment, we adapted VirulentHunter’s output by selecting its highest scoring VF class as prediction and excluding instances it classified as non-VFs (score < 0.5). DeepVIC generally outperformed VirulentHunter across most of the 14 VF classes. While VirulentHunter achieved slightly higher F_1_ scores in a few specific classes such as “immune modulation,” “biofilm,” and “nutritional/metabolic factor,” DeepVIC demonstrated superior overall performance with higher accuracy, macro, and weighted average F1 scores (Table 3), highlighting its competitiveness against SOTA models.

**Table 3:** Comparative performance of the DeepVIC multiclass classifier against VirulentHunter on the independent dataset.

Metric	DeepVIC	Virulent Hunter^a^
Accuracy	83.8	82.5
Weighted Average F1	0.839	0.828
Macro Average F1	0.758	0.721
F1 score by VF class		
Adherence	0.83	0.8156682
Invasion	0.529	0.51612903
Effector delivery system	0.88	0.86465683
Motility	0.848	0.82526316
Exotoxin	0.848	0.79889807
Exoenzyme	0.86	0.63265306
Immune modulation	0.848	0.88857546
Biofilm	0.46	0.53164557
Nutritional/Metabolic factor	0.796	0.82702703
Stress survival	0.875	0.67857143
Regulation	0.78	0.85714286
Post-translational modification	0.8	0.47619048
Antimicrobial activity/Competitive advantage	0.545	0.8
Others	0.712	0.57731959

a Sequences that failed binary classification (i.e. predictive score < 0.5) were excluded.

### 3.4 Evaluation of DeepVIC on curated VFs

To evaluate the generalizability of DeepVIC on putative VFs, two curated VF datasets of *S. pneumoniae* and *Lactoccocus* spp. were analyzed. The recall rates of DeepVIC were compared with those of BLASTP and the 6 VF predictors after excluding VFs present in the training dataset. In total, 112 putative VFs from *S. pneumoniae* and 63 from *Lactococcus* spp. were evaluated using recall because these datasets contained only VF sequences. Therefore, these analyses should be interpreted as sensitivity-oriented evaluations rather than complete performance benchmarks, as precision, specificity, MCC, AUROC, and AUPRC cannot be calculated without matched non-VF sequences.

In the *S. pneumoniae* dataset, using a cutoff of 80% identity, 70% query coverage, and an *E*-value of 1e − 10, BLASTP achieved a recall rate (19.6%, 22 out of 112), which increased to 41.1% (46 out of 112) at a lowered identity cutoff of 40%. Among the VF predictors, MP4 achieved the highest recall rate (84.8%, 95 out of 112) on the *S. pneumoniae* dataset, while DeepVIC binary model recalled 47.3% (53 out of 112) of VF sequences, performing comparably to FunGeneTyper and slightly outperforming VirulentHunter (Table 4).

**Table 4:** Recall rate comparison between DeepVIC, BLASTP and state-of-the-art VF predictors on two curated VF datasets from *Streptococcus pneumoniae* and *Lactococcus* spp.

Curated VF dataset	*Streptococcus pneumoniae*	*Lactococcus* spp.
Total sequences	150	68
Evaluated sequences	112	63
Method (Cutoff)	Total sequences recalled (recall rate)	
DeepVIC (optimal Youden’s J)	53 (47.3%)	45 (71.4%)
MP4 (0.5)	95 (84.8%)	54 (85.7%)
VirulentHunter (0.5)	47 (42.0%)	47 (74.6%)
DTVF (0.5)	62 (55.4%)	45 (71.4%)
DeepVF (0.85)	49 (43.8%)	33 (52.4%)
VirulentPred 2.0 (n/a)	68 (60.7%)	50 (79.4%)
FunGeneTyper (n/a)	53 (47.3%)	56 (88.8%)
BLASTP (80% identity, 70% query coverage)	22 (19.6%)	3 (4.76%)
BLASTP (40% identity, 70% query coverage)	46 (41.1%)	20 (31.7%)

For *Lactococcus* spp., a genus absent from the training dataset, FunGeneTyper had the highest recall rate (88.8%, 56 out of 63). DeepVIC demonstrated strong generalizability, achieving a competitive recall of 71.4% (45 out of 63), comparable to DTVF and other SOTA tools (Table 4). Notably, DeepVIC successfully identified a substantial number of VFs that BLASTP missed.

To further assess DeepVIC’s generalizability, we applied it to 73 *S. pneumoniae* serogroup 24 genomes from a previous study (Cao *et al*. 2021). DeepVIC recalled a mean of 53.9% (95% CI: 53.5–54.4%) of BLASTP hits at 80% identity, 70% query coverage, and an *E*-value of 1e − 10, accounting for a mean of 25.0% (95% CI 24.9–25.1%) of proteins as VFs per proteome. In the proteins of each serogroup 24 genome, DeepVIC predicts similar percentage of proteins as VFs and recalls comparable percentage of BLASTP hits against the curated *S. pneumoniae* VFs, demonstrating good generalizability.

Overall, DeepVIC showed competitive recall on the two curated positive-only VF datasets, although it did not achieve the highest recall in either dataset. MP4 achieved the highest recall on the *S. pneumoniae* dataset, whereas FunGeneTyper achieved the highest recall on the *Lactococcus* spp. dataset. Because these datasets are positive-only, higher recall does not necessarily indicate better overall screening performance, as false-positive predictions cannot be assessed. DeepVIC uses a cutoff optimized to balance sensitivity and specificity, which may reduce recall in positive-only evaluations but is more appropriate for balanced genome-wide VF screening.

### 3.5 Model interpretability

To identify which features drive DeepVIC’s predictions, we performed SHAP analysis across all test samples. Among the 6 PSSM-derived evolutionary features, D-FPSSM (dipeptide composition from PSSM) contributed most strongly to multiclass predictions (mean |SHAP| = 0.124), followed by PSSM-composition (0.098) and RPM-PSSM (0.087) (Fig. S4A). Notably, PLM embeddings collectively contributed approximately 60% of total feature importance, confirming that contextual sequence information is the primary driver of VF identification. Class-specific feature importance heatmap showing normalized contribution of each PSSM feature to the 14 VFDB classes. Exotoxins rely primarily on D-FPSSM (0.156), adherence factors on RPM-PSSM (0.112), and effector delivery systems on PSSM composition (0.105) (Fig. S4B). Critically, these important features correlate with known biological sequence patterns. Analysis of high-SHAP sequences for the D-FPSSM feature revealed overrepresented dipeptide motifs including C-X-C (cysteine motifs, *P* = 2.3 × 10⁻⁸), H-X-H (metal-binding motifs, *P* = 1.7 × 10⁻⁶), and G-X-G (nucleotide-binding motifs, *P* = 3.1 × 10⁻⁵) (Fig. S4C). These motifs correspond to known functional sites in bacterial toxins and adhesins, confirming that DeepVIC has learned biologically relevant patterns rather than spurious correlations.

To investigate DeepVIC’s interpretability, the mucin-binding protein (MucBP) domain was selected as a case study. MucBP domains enable lactic acid bacteria to adhere to host intestinal mucus (Boekhorst *et al*. 2006), facilitating virulence. Two putative MucBP domain-containing proteins from *L*. *graviae* (WP_014025352 and WP_014024830) were analyzed, with predicted structures and domain architectures illustrated in Fig. S5. BLASTP revealed their low similarities to *Listeria monocytogenes* Internalin J (*InlJ*) in the training dataset (WP_014025352: 47.9% identity, 64% coverage; WP_014024830: 32.6% identity, 77% coverage). Both sequences will be missed by BLASTP with most accepted cutoffs, but were correctly predicted as VFs by the binary classifier and assigned to the “adhesion” class by the multiclass classifier of DeepVIC. Shapley Additive exPlanations (SHAP) values were adopted to explore model explanations.

**Figure 6:**
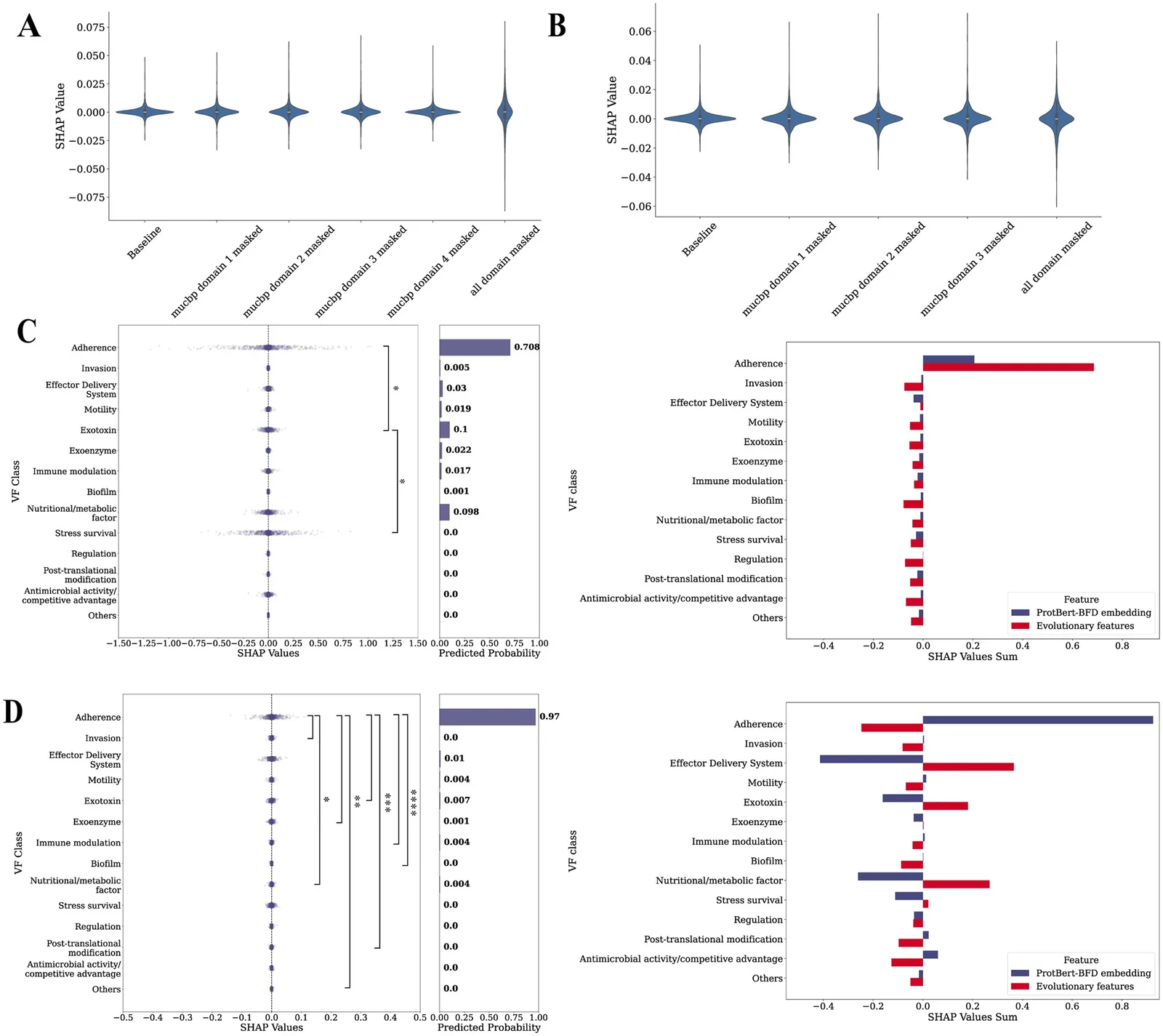
Feature importance of MucBP domain perturbations. (A, B) Distributions of SHAP values from binary classifier for WP_014025352 and WP_014024830, respectively. (C, D) Predicted probabilities and feature contribution in the multiclass model across 14 VF classes for WP_014025352 and WP_014024830, respectively.

Masking the MucBP domains in WP_014025352 and WP_014024830 reduced SHAP value distributions compared to unmasked sequence (Fig. 6A and B), indicating the potential recognition of VF-related motifs by the binary classifier model. The final predicted score and SHAP sums values decreased when MucBP domains were masked, with the lowest values observed when all MucBP domains were masked (Table 5). The model’s expected value (prediction score-SHAP value sum) aligns well with the selected cutoff of 0.537 from maximizing Youden’s *J* statistic in the 5-fold cross-validation, also suggesting that the neural network recognizes MucBP as a virulence-related domain. For the multiclass classifier, SHAP values for WP_014025352 and WP_014024830 across 14 VF classes were statistically distinct (WP_014025352.1: H = 55.66, *P *< .001, WP_014024830: *H* = 29.07, *P *< .001) and revealed a strong signal for the ground truth class “adhesion” (Fig. 6C and D). SHAP values corresponding to PLM features and evolutionary features of both sequences contributed to final predictions, but with varying dominance between them. This underscores the benefit of incorporating evolutionary features, enhancing predictive power and aligning with feature selection results.

**Table 5:** Predictive scores and corresponding SHAP values for MucBP-domain proteins following domain perturbation tests.

Sample	Perturbation	Predictive Score	SHAP value sum	Predictive score—SHAP value sum
WP_014025352	Baseline	0.996	0.467	0.530
	MucBP domain 1 Masked	0.953	0.423	0.530
	MucBP domain 2 Masked	0.965	0.435	0.530
	MucBP domain 3 Masked	0.959	0.429	0.530
	MucBP domain 4 Masked	0.987	0.457	0.530
	All MucBP domains masked	0.810	0.280	0.530
WP_014024830	Baseline	0.994	0.464	0.530
	MucBP domain 1 Masked	0.955	0.425	0.530
	MucBP domain 2 Masked	0.916	0.387	0.530
	MucBP domain 3 Masked	0.899	0.369	0.530
	All MucBP domains masked	0.294	−0.236	0.530

### 3.6 Implementation of DeepVIC

To facilitate VF research, DeepVIC has been compiled into an open-source tool and is freely available at https://github.com/ansontwk/DeepVIC. To maximize usability, we provide both a command-line implementation and a graphical user interface (GUI, Fig. S6), catering to users with varying levels of technical expertise.

## 4 Discussion

In this study, we present DeepVIC, a novel framework for VF prediction and classification that leverages multimodal features derived from PLM embeddings and evolutionary features extracted from PSSMs with a task-separation strategy, unlike existing tools that focus primarily on either VF identification or classification, or performs identification and classification in a single step. This strategy reduces model complexity and mitigating overfitting risks common in deep learning models, particularly with high-dimensional data (Lever *et al*. 2016). Furthermore, while fine-tuning pre-trained PLM for specific tasks often exacerbates overfitting (Detlefsen *et al*. 2022, Zhou *et al*. 2024), DeepVIC circumvents this issue by using PLMs solely for feature extraction rather than fine-tuning. Although this approach may yield lower performance compared to fine-tuned models (Schmirler *et al*. 2024), it significantly improves robustness and generalizability, especially for smaller datasets. Rigorous evaluation across validation and independent test sets demonstrated concordant AUROC and F_1_ scores for both binary and multiclass classifiers, confirming DeepVIC’s minimal overfitting.

Sequence redundancy and class imbalance are important considerations in VF prediction benchmarks. To evaluate the effect of redundancy, we re-trained DeepVIC using CD-HIT clustering thresholds from 70% down to 30%. As expected, stricter clustering reduced dataset size and performance, but DeepVIC retained AUROC = 0.918 and MCC = 0.676 even at 30% identity, despite retaining only 24.3% of sequences. This suggests that DeepVIC is not simply memorizing redundant homologs, although homologous information may contribute modestly to performance. We also evaluated imbalanced VF: non-VF training ratios from 1:1 to 1:20. These analyses support the use of balanced training for the final model to prevent majority-class dominance, while highlighting the importance of validation-based threshold calibration when applying DeepVIC to datasets with different VF prevalence.

Class imbalance in VF classification skews model performance, as predictions are biased toward well-represented classes (Figueroa *et al*. 2012, Rajput *et al*. 2023). In our multiclass training set, this imbalance was extreme: the “effector delivery system” class comprised 32.8% of labeled sequences, while “antimicrobial activity/competitive advantage” represented only 0.18%. Analysis of inter- and intra-class cosine similarity on DeepVIC’s training dataset revealed that underrepresented VF classes with fewer sequences often exhibit higher similarity, potentially inflating performance metrics due to overlap between training and test subsets. To mitigate this, DeepVIC leverages the synthetic minority oversampling technique (SMOTE), which generates synthetic samples for minority classes rather than duplicating existing data. This approach avoids overfitting risks associated with data leakage while preserving dataset integrity. Alternative strategies, such as class weighting, have shown inconsistent results (Zheng *et al*. 2020). However, the use of SMOTE is particularly well-suited to our framework because DeepVIC operates on continuous, dense ProtBert-BFD embeddings rather than sparse categorical inputs. In such a representation space, feature-space interpolation between neighbouring minority samples preserves meaningful similarity structure and produces synthetic examples that are more biologically plausible than simple duplication (Chawla *et al*. 2002). SMOTE substantially improved the macro-average F1 score and prevented the model from biasing predictions toward majority classes. However, performance on the smallest classes (“invasion,” “biofilm”) remained modest (F1 < 0.55, Table 2), indicating that synthetic samples cannot fully compensate for fundamental data scarcity and inherent functional ambiguity. Similar observations have been reported in other biological classification tasks using PLM embeddings (Chamseddine *et al*. 2022, Liu and Tian 2023, Nafi 2025, Shoombuatong *et al*. 2025), suggesting that targeted experimental curation of underrepresented VF classes remains essential. We therefore view SMOTE as a strategy for mitigating class imbalance rather than a replacement for collecting additional minority-class data.

Most of the current VF classifiers, including DeepVIC, assume that VFs are exclusive to a single functional class. However, many VFs are multifunctional, with roles that vary across host-pathogen environments (Qin *et al*. 2022, Sharma *et al*. 2022). For example, *Streptococcus* pilus proteins are categorized as ‘adherence’ in VFDB but also contribute to invasion in reality (Maisey *et al*. 2007, Sharma *et al*. 2013), highlighting the limitation of mutually exclusive class definitions. This complexity is further evidenced by the overlapping clusters observed in UMAP projections of VF data in this study (Fig. 2A and B). While PLM embeddings and evolutionary features provide valuable multimodal features, they do not fully resolve ambiguities in classifying functional overlapping VFs. The comparison between ProtBert-BFD and ISM embeddings indicates that PLM selection can influence VF classification, but neither representation was statistically superior under our evaluation. ISM achieved competitive class-specific performance, suggesting that structurally enriched embeddings may provide useful information for certain VF categories. However, ProtBert-BFD combined with six selected evolutionary features achieved the best aggregate performance and the best class-specific F1 score in 7 out of 14 VF classes, including several large classes. ProtBert-BFD also allowed longer sequence coverage under our implementation, reducing truncation for large multidomain VF proteins. Therefore, ProtBert-BFD was selected as the default representation based on comparable performance, practical sequence-coverage advantages, and consistency across DeepVIC modules rather than statistically significant superiority over ISM. Future investigations in VF classification should place more emphasis on exploring multi-label classification, which has been successfully applied in protein localization predictions and functional annotations (Gligorijević *et al*. 2021, Wang and Wei 2022). Additionally, while VFDB proposes subclass-level classification, its implementation remains challenging due to the inherent diversity of known VFs and limited sequence availability for many subclasses. The combination of high sequence similarities among related VFs and sparse subclass representation creates substantial risks of overfitting and reduced generalizability. Consequently, DeepVIC currently operates at the level of 14 VFDB classes, leaving subclass classification as an important avenue for further investigation.

Model interpretability remains a critical challenge in deep learning applications for biological sequence analysis (Azodi *et al*. 2020, Ji *et al*. 2023, Sun *et al*. 2024, Zhang *et al*. 2024). DeepVIC addresses this gap by demonstrating how functionally critical protein domains influence prediction outcomes through comprehensive interpretability analysis. Our framework employs SHAP values to quantify feature importance, revealing that the sum of SHAP values for query samples closely approximates the difference between predicted outputs and the expected baseline (average prediction for VFs and non-VFs prior to feature input) (Lundberg and Lee 2017). This strong concordance validates our classification approach, with true VF predictions consistently exhibiting net positive SHAP values while non-VFs show the opposite pattern. We acknowledge that SHAP-based analysis provides interpretability by quantifying feature contributions, but this does not constitute full model explainability. While our perturbation analysis identified the MucBP domain as influential for adherence predictions, causal validation of these computational findings requires experimental follow-up. Therefore, we describe DeepVIC as offering enhanced interpretability rather than being a fully explainable model. Our extended interpretability analysis reveals that DeepVIC’s predictions are grounded in biologically meaningful features. The D-FPSSM feature, which captures evolutionarily constrained dipeptide patterns, emerged as the most important PSSM-derived contributor—particularly for exotoxin and effector delivery predictions. The identification of overrepresented C-X-C and H-X-H motifs in high-SHAP sequences is striking, as these correspond to known functional signatures: cysteine motifs are critical for toxin structure stabilization, while histidine motifs often coordinate metal ions in active sites. This alignment between computational feature importance and established biochemistry suggests that DeepVIC has learned genuine virulence-associated patterns rather than dataset-specific artifacts. However, we caution that SHAP values provide correlative rather than causal insights; experimental validation remains the gold standard for confirming predicted functional motifs.

We benchmarked DeepVIC models against 6 SOTA predictors using both fair retraining and external curated-dataset evaluations. In the fair retraining benchmark, all available comparator models were retrained or reimplemented using the same training dataset and evaluated on the same independent test set. Under this setting, DeepVIC achieved the highest F1-score, MCC, AUROC, precision, and recall among the evaluated models, although the DeepVF-style stacked model achieved higher accuracy and specificity. This suggests that DeepVIC provides strong overall discriminative performance, whereas different tools may be preferable depending on whether sensitivity or specificity is prioritized. The two literature-curated VF datasets provide useful evidence for cross-species sensitivity but have important limitations. Because they contain only positive VF sequences, performance can only be assessed by recall. A method that predicts more sequences as VFs may achieve higher recall but could also generate more false positives in genome-wide screening. This explains why tools such as MP4 and FunGeneTyper may achieve higher recall than DeepVIC on the curated datasets, while not necessarily providing better balanced performance. For applications prioritizing maximum sensitivity, such tools may be preferred. In contrast, DeepVIC is designed for balanced VF identification together with VFDB-aligned multiclass classification and interpretability.

In addition, we provide DeepVIC as an open-source program with a graphical user interface, enabling reliable, local execution for researchers regardless of their computational expertise.

In conclusion, despite the inherent complexities of VF classification, sequence redundancy, and data imbalance, DeepVIC demonstrates robust and balanced performance through its modular multimodal framework. The methodologies developed, including our perturbation analyses and cutoff optimization strategies, show promising potential for transfer to other protein prediction tasks such as antimicrobial resistance prediction. This work establishes a strong foundation for future advancements in computational analysis of VF and related protein families.

## Supplementary Material

vbag237_Supplementary_Data

## Data Availability

Source material, including code and data, and implementation DeepVIC are available at https://github.com/ansontwk/DeepVIC.
