# Supplementary material for: DeepVIC: modular prediction and classification of bacterial virulence factors using protein language model embeddings
Source: Bioinform Adv. 2026 Aug 17;6(1):vbag237. doi: 10.1093/bioadv/vbag237 (PMC13539359; doi:10.1093/bioadv/vbag237)
Supplement: Supplementary file 1 [file vbag237_supplementary_data.zip › Supplementary Figures_2e.docx]

**Supplementary Figures**

 **Supplementary Figure S1** (A) Dataset curation and cleaning workflow. (B) Schematic non-VFDB virulence factor sequence labelling workflow.

**Supplementary Figure S2** Schematic overview of the two-stage optimization strategy and evaluation of the DeepVIC multiclass model.

**Supplementary Figure S3** VF sequence (A) class label distribution, (B) length distribution, (C) family and (D) genus distribution. The 20 most-occurring taxa is presented. Scatter plots for VF class-specific metrics: (E) number of labelled sequences in dataset against mean cosine similarity, (F) mean cosine similarity against F_1_ scores, and (G) number of labelled sequences in dataset against F_1_ scores.

**Supplementary Figure S4 DeepVIC interpretability analysis.** (A) Global feature importance showing mean |SHAP| values for PLM embeddings (aggregate) and six PSSM-derived evolutionary features. Error bars represent standard deviation across 5-fold cross-validation. D-FPSSM (dipeptide composition from PSSM) is the most important PSSM-derived feature (0.124). (B) Class-specific feature importance heatmap showing normalized contribution of each PSSM feature to the 14 VFDB classes. Exotoxins rely primarily on D-FPSSM (0.156), adherence factors on RPM-PSSM (0.112), and effector delivery systems on PSSM composition (0.105). (C) Motif discovery from sequences with highest D-FPSSM SHAP values. Overrepresented motifs include C-X-C (cysteine, p=2.3×10⁻⁸), H-X-H (histidine, p=1.7×10⁻⁶), and G-X-G (glycine, p=3.1×10⁻⁵), corresponding to known functional sites in bacterial toxins, metalloproteins, and nucleotide-binding domains.

**Supplementary Figure S5** Predicted structures of mucin-binding protein (MucBP) domain-containing proteins (A) WP_014025352 and (B) WP_014024830 respectively. Red regions represent MucBP domains. (C) The predicted motifs of the two MucBP domain containing proteins.

**Supplementary Figure S6** The standalone and user-friendly graphical user interface of DeepVIC for local execution.
