# Supplementary material for: DeepVIC: modular prediction and classification of bacterial virulence factors using protein language model embeddings
Source: Bioinform Adv. 2026 Aug 17;6(1):vbag237. doi: 10.1093/bioadv/vbag237 (PMC13539359; doi:10.1093/bioadv/vbag237)
Supplement: Supplementary file 1 [file vbag237_supplementary_data.zip › Supplementary Methods_v2.docx]

**Supplementary Methods**

**Supplementary Method S1 *Protein sequence one-hot encoding***

Protein sequences were converted into one-hot matrices, corresponding to 20 natural occurring amino acids, “X” for ambiguous or uncommon amino acids (“U”, “B”, “Z”, “O” and “J” ) and a padding dimension for sequences shorter than that of 2000 amino acids. Sequences longer than 2000 amino acids were truncated. For each input sequence, a one-hot matrix of size 22 x 2000 was generated.

**Supplementary Method S2 *Additional robustness and benchmarking analyses***

**Clustering-threshold sensitivity analysis.**

To evaluate whether sequence redundancy influenced binary classifier performance, we re-trained DeepVIC using datasets clustered at CD-HIT identity thresholds of 30%, 40%, 50%, 60%, and 70%. For each threshold, representative sequences were retained and the same DeepVIC binary architecture and hyperparameters were used. Model performance was evaluated using AUROC, F1-score, accuracy, and MCC. The results are reported in Supplementary Table S10.

**Imbalance-sensitivity analysis**

To assess whether balanced training datasets reflect realistic VF screening, we performed imbalance-sensitivity analyses using VF:non-VF ratios of 1:1, 1:2, 1:5, 1:10, and 1:20. Models were trained using the same DeepVIC binary architecture and evaluated on a fixed independent test set. Validation-based threshold calibration was also assessed to examine whether decision-threshold adjustment could recover balanced performance under different class distributions.

**Benchmarking against traditional machine learning and neural network models**

To benchmark DeepVIC against alternative modeling strategies, we compared it with XGBoost, Random Forest, SVM, and 1D-CNN using the same dataset split and input feature representation. Model performance was evaluated using accuracy, F1-score, AUROC, MCC, and training time.

**Fair retraining benchmark of SOTA predictors**

To address potential bias caused by comparing models trained on different datasets, we retrained or reimplemented available SOTA VF predictors using the same training dataset used for DeepVIC and evaluated them on the same independent test set. The retrained/reimplemented methods included MP4, DTVF, FunGeneTyper-style embedding classifier, DeepVF-style stacked model, VirulentHunter, and VirulentPred-style AutoGluon model. For methods that generated prediction probabilities, the classification threshold was optimized using the validation set where applicable and then applied to the independent test set. Performance was assessed using accuracy, precision, recall, specificity, F1-score, MCC, and AUROC.

**Supplementary Method S3 *Protein structure prediction and visualization***

ESMFoldv1 [1] was used to predict the structure of the two MucBP domain protein selected in the interpretability analysis and were visualized using open source PyMOL (version 3.0.0) (<https://github.com/schrodinger/pymol-open-source>) [2]. Protein domain architecture figures were produced using drawProtein (version 1.22.2) [3] in R (version 4.3.2).

**Supplementary Reference**

1.	Lin, Z., et al., *Evolutionary-scale prediction of atomic-level protein structure with a language model.* Science, 2023. **379**(6637): p. 1123-1130.

2.	Schrödinger, L., *The PyMOL Molecular Graphics System, Version 3.0*.

3.	Brennan, P., *drawProteins: a Bioconductor/R package for reproducible and programmatic generation of protein schematics.* F1000Res, 2018. **7**: p. 1105.
